# Micro-Vibration Control of Deployable Space Optical Imaging System Using Distributed Active Vibration Absorbers

**DOI:** 10.3390/s25040989

**Published:** 2025-02-07

**Authors:** Zhuo Chen, Guangyuan Wang, Chuanwen Zhu, Feihu Liu, Kuai Yu, Yongsheng Wu

**Affiliations:** China Academy of Space Technology, Beijing 100080, China; chenzhuo183@mails.ucas.edu.cn (Z.C.);

**Keywords:** micro-vibration, distributed control, inertial absorber, LMS algorithm

## Abstract

This paper presents a distributed vibration control method using attachable absorbers for micro-vibration control of large space payload structures. The distributed vibration control system is modeled at three levels. The simplification of the attachable absorber model is discussed, and the single-channel ANC controller is extended to a multi-channel configuration. Based on the models, simulation analysis is conducted, revealing that the voltage–force output of the absorber in the low-frequency range can be simplified to a second-order system. During the distributed vibration control system simulation, a Simulink–GA hybrid optimization is applied to address the large number of converging parameters. The optimized parameters successfully control the vibration of all channels. Further analysis indicates that the coupling between control channels slightly reduces convergence speed but has no impact on the final control effect. Additionally, the control system can achieve the same results by independently tuning parameters for each channel. The experimental results, using absorber prototypes and a model with 12 sub-mirror structures, demonstrate that the method can control sub-mirror vibrations simultaneously, maintaining the flatness of the main mirror under disturbance, with a 90% reduction in vibration within 4 s. The coupling effect reduces the final convergence speed by approximately 10%, with a time difference of around 1 s.

## 1. Introduction

Spaceborne optical remote sensors are less affected by atmospheric disturbances and are not restricted by geographical location, playing a crucial role in fields such as Earth observation and astronomical exploration. Segmented deployable imaging systems can overcome launch capacity limitations and achieve larger apertures compared to monolithic imaging systems [1,2]. However, various micro-vibrations in the space environment significantly limit the ability of optical remote sensors to achieve precise image stabilization [3,4,5]. Disturbances originating from the satellite body are typically mitigated through multi-stage passive and active vibration isolation systems, but the complex internal structures of optical payloads render vibration isolation platforms ineffective. It is necessary to employ flexibly arranged devices, which can control multi-point vibrations [6,7,8,9]. A distributed vibration control approach is required to achieve overall structural vibration suppression effectively.

The concept of distributed control has a long history, initially emerging to address the insufficient vibration control capabilities of large-scale structural systems and primarily focusing on the placement of sensors and actuators to balance control effectiveness and cost. For instance, Hanagan et al. [10] optimized the placement of sensors and actuators and the feedback gain coefficients for vibration control of lightweight floors. Similarly, Pereira et al. [11] further optimized vibration control for indoor walkway structures by introducing high-pass/low-pass filters to improve the velocity feedback signal derived from the acceleration signal. Camacho-Gómez et al. [12] demonstrated the effectiveness of novel metaheuristic algorithms, such as Coral Reefs Optimization, for optimizing actuator–sensor (A/S) placement in floor vibration control. Cao et al. [13] developed a dynamic model for T-shaped bending–torsion structures and optimized actuator placement to minimize input energy. Swanson et al. [14] introduced a circumferential force generator (CFG) developed by LORD Corporation to compensate for imbalance-induced disturbances from helicopter rotor blades. They tested the vibration control of a helicopter fuselage model with multiple CFGs placed at different positions. Tang et al. [15] employed cable actuators to control the vibration of membrane diffraction telescopes and optimized the placement of different numbers of actuators using particle swarm optimization.

With the advent of new materials, the introduction of novel structural concepts, and breakthroughs in computational power, the algorithms and controller designs for distributed control have advanced rapidly. Early research by Tzou et al. [16] investigated vibration control of a sandwich beam structure with piezoelectric ceramic (PZT) layers on both sides (designated as S-layer and A-layer). Burke et al. [17] addressed vibration control for simply supported beams, designing a controller described in both spatial and temporal coordinates. Berkhoff et al. [18,19] improved the multi-input multi-output (MIMO) filtered-error algorithm for multi-channel noise control and applied this algorithm to the design of high-authority controllers (HAC) paired with proportional feedback low-authority controllers (LAC) to work for printed circuit board (PCB) vibration control. Kumar et al. [20] used PZT actuators to control vibrations in L-shaped beams. They employed a neural network to classify beams based on their first two natural frequencies, facilitating state-space and controller updates for slowly varying systems.

Li et al. [21] designed vibration control for large tensile structures by dividing the overall structure into substructures and designing output feedback controllers for each using μ-synthesis theory. Ferrari et al. [22] investigated vibration control of a composite plate using four pairs of Macro Fiber Composite (MFC) actuators and sensors with non-parallel Positive Position Feedback (PPF) control. Omidi et al. [23] proposed an Integrated Consistency Controller (ICC) for the distributed control of flexible structures. By introducing an integral penalty term for state variable differences between subunits, the ICC improved fault tolerance against sensor or actuator failures. Wang et al. [24,25] applied a similar concept to solar array vibration control, dividing the array into subunits along its length. The method was extended to on-orbit assembly of solar array structures, where the state-space and controller models were updated as subunits were assembled. Li et al. [26] improved the MIMO direct vibration control method based on the system’s frequency response matrix by incorporating an update mechanism for the impedance matrix alongside error-variable step-size updates for control signals. Xie et al. [27] proposed a controller design method based on system identification data, reconstructing and expanding the target system’s transfer function matrix from time-domain signals of channel outputs and responses. Ji et al. [28] developed an observer for vibration control of a central rigid body structure flanked by flexible beams and designed a distributed force feedback controller based on these observations.

However, the control methods proposed in the aforementioned studies are mostly theoretical. Some are purely mathematical models that do not account for practical implementation, while others involve experimental testing on simple structures such as composite plates or cantilever beams. These structures have relatively straightforward theoretical models and identification processes. Additionally, actuators such as MFCs, commonly used in these studies, are currently unsuitable for application to large-scale on-orbit structures due to their operational principles. Furthermore, most algorithms or parameter determination methods rely heavily on identifying the overall model of the target structure, with some even requiring real-time updates and decomposition of the actual transfer function matrix. Large space structures exhibit significant differences between their on-orbit and ground states, and their operational modes often limit the feasibility of in-orbit identification to small localized regions. This constraint further increases the engineering challenges of applying the aforementioned methods in practical scenarios.

This paper introduces a distributed vibration control method for a primary mirror structure consisting of 12 sub-mirrors, employing an attachable absorber as the actuator. The method utilizes an improved multi-channel Adaptive Notch Canceller (ANC) controller based on the LMS algorithm. Its effectiveness is demonstrated through modeling, simulation, and experimental validation. The structure of this paper is as follows: Section 2 develops mathematical models for the system, including the attachable absorber, target structure, and closed-loop control system. It outlines the methods for constructing the simulation system, optimization strategies for parameters, and the design of a distributed vibration control experimental system for verification. Section 3 presents the simulation results, as well as further analyses and comparative simulations based on these results, and subsequently provides the experimental outcomes. Section 4 analyzes and discusses the experimental data. Finally, Section 5 concludes the study with a summary of findings.

## 2. Materials and Methods

### 2.1. System Model

**Figure 1:**
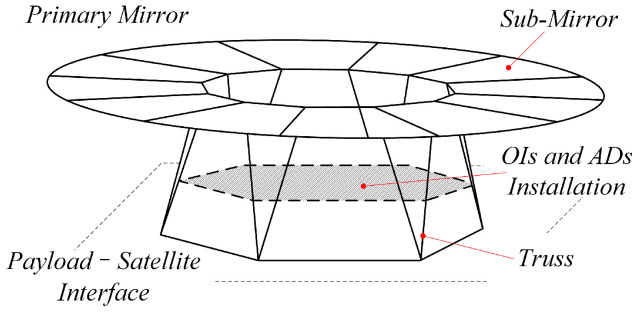
Schematics of a large deployable space optical structure.

A schematic diagram of a large deployable space optical structure is shown in Figure 1. Its functional components include the primary mirror, secondary mirror, optical instruments, and auxiliary devices (OIs and ADs). To maximize the size of the primary mirror within the constraints of limited launch capacity, the primary mirror is designed as an assembly of multiple sub-mirrors. These sub-mirrors are independently connected to the structural main body.

Evidently, the flatness of the primary mirror directly affects the final imaging quality. Under the influence of internal and external disturbances, each sub-mirror may experience individual vibrations. In particular, differences in the normal positions of the mirror surfaces can significantly degrade the flatness of the primary mirror, thereby impairing imaging quality. Therefore, vibration control is necessary. Since there is no direct interaction between the sub-mirrors, simultaneous and independent control of each sub-mirror is required.

**Figure 2:**
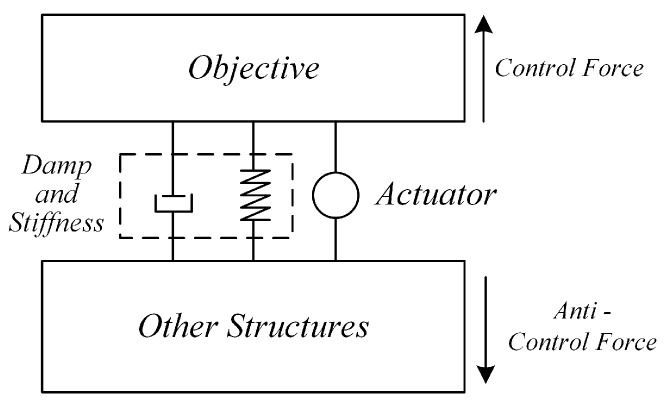
Basic principle of active vibration control with the actuator.

Applying control forces to the target structure (objective) using actuators to reduce vibrations through cancellation is a typical method of active vibration control, as illustrated in Figure 2. Depending on the requirements for control force and stroke, various actuators can be employed, such as piezoelectric materials, magnetostrictive materials, and voice coil motors. According to the principle of action and reaction, these actuators require corresponding support structures (other structures). While exerting control forces on the target structure, they also generate reactive forces (control reaction forces) that act on other parts of the system, potentially causing vibrations in those structures. In most scenarios, these “other structures” are typically vibration sources or high-mass bases, where vibrations induced by control reaction forces are either negligible or inconsequential. However, for micro-vibration control within the payloads of large on-orbit space structures, the microgravity environment and structural flexibility mean that control reaction forces can induce micro-vibrations in other parts of the payload. This can prevent overall system performance from improving as vibrations are controlled and, in some cases, even worsen it—a phenomenon known as the “waterbed effect”. To enable flexible vibration control while minimizing the impact of control reaction forces, an attachable absorber based on inertial actuation principles is employed.

This subsection develops models for the relationship between the input signal and output force of the attachable absorber, the relationship between the input force and output displacement of the target structure, and the distributed closed-loop control circuit. These models serve as the foundation for subsequent simulations and analyses.

#### 2.1.1. Model of the Attachable Absorber

**Figure 3:**
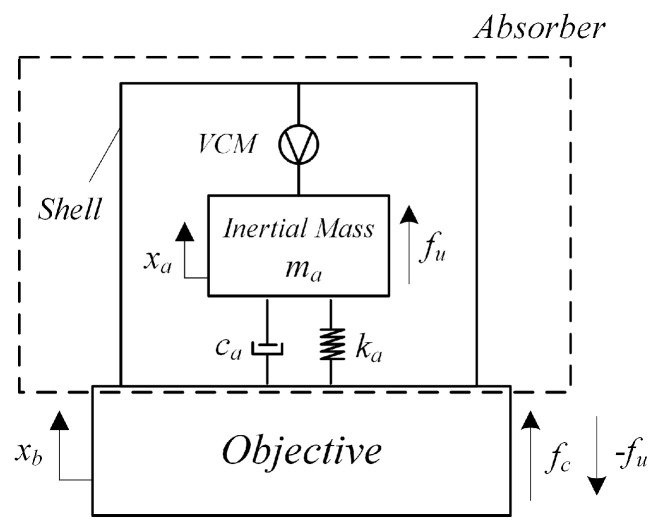
Mechanism of an attachable absorber based on the VCM.

As shown in Figure 3, the attachable absorber is mounted on the objective via its shell. Upon receiving a control voltage input, the voice coil motor (VCM) drives the inertial mass (mover) into motion, applying a control force to the objective. By conducting a force analysis on the objective depicted in the figure, we have:

(1) $$f_{c}=k_{a}\left(x_{a}-x_{b}\right)+c_{a}\left({\dot{x}}_{a}-{\dot{x}}_{b}\right)-f_{u}$$

By performing a force analysis on the mover of the attachable absorber, we have:

(2) $$m_{a}{\ddot{x}}_{a}+c_{a}\left({\dot{x}}_{a}-{\dot{x}}_{b}\right)+k_{a}\left(x_{a}-x_{b}\right)=f_{u}$$

A single dot indicates the first-order derivative with respect to time, while a double dot indicates the second-order derivative. $f_{c}$ is the control force exerted by the absorber on the objective, $x_{b}$ represents the vibration displacement of the objective, $x_{a}$ denotes the displacement of the inertial mass of the absorber, $k_{a}$ and $c_{a}$ are the stiffness and damping coefficients between the mover and the shell of the absorber, $m_{a}$ is the mass of the mover, and $f_{u}$ is the driving force generated by the VCM. Based on the operating principle of the VCM, the driving force is given by:

(3) $$f_{u}=k_{e}I_{e}$$

(4) $$u_{e}=L{\dot{I}}_{e}+RI_{e}+k_{e}\left({\dot{x}}_{a}-{\dot{x}}_{b}\right)$$

Here, $k_{e}$ represents the electromagnetic force constant of the VCM, $u_{e}$ is the input voltage to the VCM, $I_{e}$ denotes the coil current, and R and L are the resistance and inductance of the coil, respectively.

Compared to the driving force, the damping force is typically very small and can be neglected, so we assume $c_{a}=0$. Combining Equations (1) to (4) and letting $\Delta x=x_{a}-x_{b}$, we have:

(5) $$f_{c}=-m_{a}{\ddot{x}}_{a}$$

(6) $$u_{e}=\frac{Lk_{a}+k_{e}^{2}}{k_{e}}\dot{\Delta x}+\frac{Rk_{a}}{k_{e}}\Delta x-\left(\frac{L}{k_{e}}\dot{f_{c}}+\frac{R}{k_{e}}f_{c}\right)$$

Taking the Laplace transform of Equations (5) and (6), we get:

(7) $$F_{c}\left(s\right)=-\frac{m_{a}k_{e}s^{2}}{m_{a}Ls^{3}+m_{a}Rs^{2}+\left(k_{a}L+k_{e}^{2}\right)s+k_{a}R}U_{e}\left(s\right)-\frac{m_{a}\left(k_{a}L+k_{e}^{2}\right)s^{3}+m_{a}k_{a}Rs^{2}}{m_{a}Ls^{3}+m_{a}Rs^{2}+\left(k_{a}L+k_{e}^{2}\right)s+k_{a}R}X_{b}\left(s\right)$$

Equation (7) indicates that the output force of the attachable absorber is related to both the input voltage and the motion of the objective, as the objective’s motion affects the elastic force between the mover and the shell, as well as the back electromotive force in the VCM coil. The absorber generates an inertial force through the motion of the mover. When performing micro-vibration control, the magnitude of the motion of the objective is very small relative to the mover, and it further decreases with continued control, ideally approaching zero. Therefore, the influence of the objective’s motion on the output force of the absorber can be neglected, and the $X_{b}\left(s\right)$ term can be omitted. As a result, the transfer function between the input voltage of the attachable absorber and its output force is given by:

(8) $$G\left(s\right)=\frac{F_{c}\left(s\right)}{U_{e}\left(s\right)}=-\frac{m_{a}k_{e}s^{2}}{m_{a}Ls^{3}+m_{a}Rs^{2}+\left(k_{a}L+k_{e}^{2}\right)s+k_{a}R}$$

Furthermore, the literature [29] indicates that the inductance L is very small and its effect can be neglected. With this simplification, the transfer function can be further reduced to:

(9) $$G^{\prime }\left(s\right)=-\frac{m_{a}k_{e}s^{2}}{m_{a}Rs^{2}+k_{e}^{2}s+k_{a}R}$$

Equation (8) represents the model for generating control force from the voltage signal, while Equation (9) is the simplified version of the model.

#### 2.1.2. Model of the Objective

The input–output relationship of the objective is described using the second-order ordinary differential equation of a multi-degree-of-freedom dynamic system as follows:

(10) $$M\ddot{q}+D\dot{q}+Kq=B_{q}(f_{d}+f_{cu})$$

(11) $$y=C_{q}q$$

For a structure with $n_{d}$ degrees of freedom, p inputs, and o outputs, q represents the $n_{d}\times 1$ displacement vector and M, D, and K are the $n_{d}\times n_{d}$ mass matrix, damping matrix, and stiffness matrix, respectively. $B_{q}$ is the $n_{d}\times p$ input matrix, $C_{q}$ is the $o\times n_{d}$ output matrix, y is the o×1 output displacement vector, and $f_{d}+f_{cu}$ represents the p×1 force input vector, which includes the disturbance force $f_{d}$ and the control force $f_{cu}$.

Based on Equations (8) and (9), the input–output relationship between the control force $f_{cu}$ and the p×1 control voltage vector u can be described using a transformation matrix $\Gamma_{u}$, such that:

(12) $$f_{cu}=\Gamma_{u}u$$

For practical structures, the $n_{d}$ scale is typically very large, resulting in an excessively high system dimension, and the parameters M, D, and K are challenging to obtain. Therefore, the actual coordinates are usually transformed into modal coordinates and described using the relevant parameters. Let:

(13) $$q=\phi q_{m}$$

Here, $q_{m}$ represents the r×1 modal coordinate vector, ϕ is the $n_{d}\times r$ mass-normalized mode shape matrix of the structure, and r is the number of modal degrees of freedom. Substituting Equations (12) and (13) into Equations (10) and (11), and pre-multiplying Equation (10) by $\phi^{T}$, the following expression can be obtained:

(14) $$\ddot{q_{m}}+2Z\Lambda \dot{q_{m}}+\Lambda^{2}q_{m}=B_{m}\left(f_{d}+\Gamma_{u}u\right)$$

(15) $$y=C_{m}q_{m}$$

Here, $\Lambda =diag\{\omega_{1},\omega_{2},\ldots ,\omega_{r}\}$, where $\omega_{i}$ represents the i-th natural frequency of the structure, and $Z=diag\{\zeta_{1},\zeta_{2},\ldots ,\zeta_{r}\}$, where $\zeta_{i}$ is the corresponding damping ratio. Additionally, $B_{m}=\phi^{T}B_{q}$ and $C_{m}=C_{q}\phi$.

To facilitate time-domain simulation and control analysis, the system described by Equations (14) and (15) is represented in state-space form as follows:

(16) $$\begin{array}{c} \left\{\begin{array}{l} \dot{x}=Ax+B\left(f_{d}+\Gamma_{u}u\right)\\ y=Cx \end{array}\right.\\ x={\left[\begin{array}{cc} q_{m} & \dot{q_{m}} \end{array}\right]}^{T}\\ A=\left[\begin{array}{cc} 0 & I\\ -\Lambda^{2} & -2Z\Lambda \end{array}\right],\;\;\;B=\left[\begin{array}{c} 0\\ B_{m} \end{array}\right],\;\;\;C=\left[\begin{array}{cc} C_{m} & 0 \end{array}\right] \end{array}$$

#### 2.1.3. Model of the Closed-Loop Control System

In engineering applications, the primary objective of vibration control is to reduce the displacement, velocity, or acceleration of the structure, ideally bringing them to zero. These physical quantities, as system outputs, can be directly measured using corresponding types of sensors. In the state-space model from the previous section, the output displacement vector and its derivatives can be used as error inputs to the controller. The following uses displacement as an example.

Based on the operational characteristics of on-orbit equipment, an improved ANC vibration control method using a variable step-size LMS algorithm proposed in [30] is employed. According to this algorithm, the process for generating the control voltage signal $u_{j}(n)$ corresponding to the sampled output displacement $y_{j}(n)$ of the j-th degree of freedom is as follows:

(17) $$\begin{array}{c} \;\;\;\;\;\;\;\;\;\;\;\;\;\;\;\;\;\;\;\;\;\;\;\;\;\;\;\;\;\;\;\;\;\;\;\;\;\;\;\;\;\;u_{j}\left(n\right)=\omega_{jsin}\left(n\right)\sin\left(2\pi f_{ctr}n\right)+\omega_{jcos}\left(n\right)\cos\left(2\pi f_{ctr}n\right)\\ \;\;\;\;\;\;\;\;\;\;\;\;\;\;\;\;\;\;\;\;\;\;\;\;\;\;\;\;\;\;\omega_{jsin}\left(n+1\right)=\omega_{jsin}\left(n\right)-2\mu_{j}\left(n\right)y_{j}\left(n\right)\rho_{j}\sin\left(2\pi f_{ctr}n+\varphi_{j}\right)\\ \;\;\;\;\;\;\;\;\;\;\;\;\;\;\;\;\;\;\;\;\;\;\;\;\;\;\;\;\;\;\omega_{jcos}\left(n+1\right)=\omega_{jcos}\left(n\right)-2\mu_{j}(n)y_{j}(n)\rho_{j}\cos\left(2\pi f_{ctr}n+\varphi_{j}\right)\\ \mu_{j}\left(n\right)=\alpha_{j}+\beta_{j}\left(1-e^{-{y_{j}}^{2}\left(n\right)}\right) \end{array}$$

In the equation, n denotes the sampling sequence, $f_{ctr}$ represents the target control frequency, $\omega_{jsin}\left(n\right)$ and $\omega_{jcos}\left(n\right)$ are the weight coefficients of the reference signals, $\rho_{j}$ and $\varphi_{j}$ are the gain and delay introduced by the secondary path, $\mu_{j}\left(n\right)$ is the convergence iteration factor, and $\alpha_{j}$ and $\beta_{j}$ are the fixed and variable step-size coefficients, respectively.

Equation (17) can be expressed in the following vector form:

(18) $$\begin{array}{l} \;\;\;\;\;\;\;\;\;\left\{X\left(n\right)\right\}={\left[\sin\left(2\pi f_{ctr}n\right),\cos\left(2\pi f_{ctr}n\right)\right]}^{T}\\ \;\;\;\;\;\;\;\;\;\left\{Y_{j}\left(n\right)\right\}={\left[\rho_{j}\sin\left(2\pi f_{ctr}n+\varphi_{j}\right),\rho_{j}\cos\left(2\pi f_{ctr}n+\varphi_{j}\right)\right]}^{T}\\ \;\;\;\;\;\;\;\;\;\left\{W_{j}\left(n\right)\right\}={\left[\omega_{jsin}\left(n\right),\omega_{jcos}\left(n\right)\right]}^{T}\\ \left\{W_{j}\left(n+1\right)\right\}=\left\{W_{j}\left(n\right)\right\}-2\mu_{j}\left(n\right)y_{j}\left(n\right)\left\{Y_{j}\left(n\right)\right\}\\ \;\;\;\;\;\;\;\;\;\;\;\;\;u_{j}\left(n\right)={\left\{W_{j}\left(n\right)\right\}}^{T}\left\{X\left(n\right)\right\}\\ \;\;\;\;\;\;\;\;\;\;\;\;\;\mu_{j}\left(n\right)=\alpha_{j}+\beta_{j}\left(1-e^{-{y_{j}}^{2}\left(n\right)}\right) \end{array}$$

**Figure 4:**
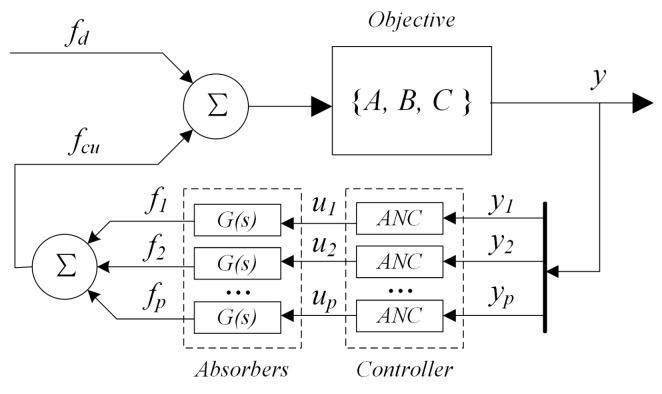
Distributed vibration control close-loop model.

By simultaneously applying the aforementioned controller to the outputs of multiple degrees of freedom, distributed vibration control for the entire structure can be achieved. Incorporating the above elements, the overall closed-loop control model can be represented as shown in Figure 4.

This control method represents a decoupled control approach, treating the multi-input multi-output (MIMO) problem of controlling multi-point vibrations of the objective as multiple independent single-input single-output (Multi-SISO) control loops. It neglects the coupling relationships between different control channels, meaning that the influence of the control outputs from other channels on the error of a specific channel is not considered when controlling that channel. According to the convergence conditions of the LMS algorithm, the convergence factor must satisfy:

(19) $$0<\mu \left(n\right)<\frac{1}{\lambda_{jmax}}$$

This ensures that the weight coefficient iterations lead to control convergence. $\lambda_{jmax}$ is the maximum eigenvalue of the autocorrelation matrix of the single-channel signal $\left\{Y_{j}\left(n\right)\right\}$. In theory, as long as the structure remains unchanged, this value is not influenced by other control channels. However, since the optimal weight coefficients for a single signal during convergence are related to the error signal, the convergence process may be affected.

### 2.2. Numerical Simulation System

#### 2.2.1. Establishment of Simulation

By performing finite element analysis on the objective, information such as modal mass and mode shapes can be obtained. After modal truncation, the state-space model described in Equation (16) can be established. For this simulation, the modal shapes corresponding to the natural frequencies within 300 Hz are selected for modeling, resulting in a total of 108 modes. The lowest natural frequency is 24.96 Hz, and the highest is 296.84 Hz. The model includes 13 input degrees of freedom and 12 output degrees of freedom. The 13 input degrees of freedom consist of one disturbance force input and 12 control force inputs. The disturbance force is a sinusoidal excitation with a frequency of 42 Hz and an amplitude of 20 N, applied at the center of the OIs and ADs installation area, as shown in Figure 1. The 12 control force inputs and displacement output positions correspond to the centers of the 12 sub-mirrors, all oriented in the normal direction of the primary mirror surface. After obtaining the state-space model, the simulation system is constructed in Simulink, with a Runge–Kutta solver and a simulation step size of 0.001 s.

**Figure 5:**
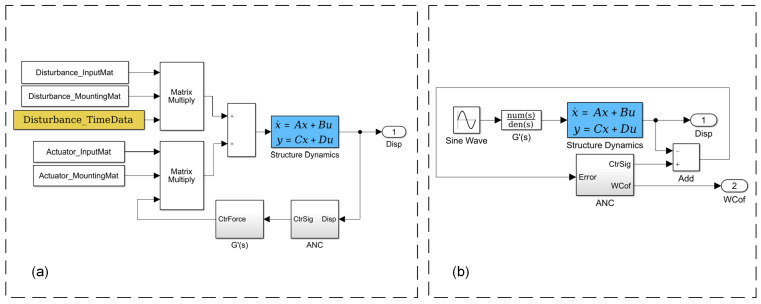
Simulation system: (**a**) distributed closed-loop control; (**b**) identification for single channel.

Figure 5a shows the construction of the distributed closed-loop control system, where the input/output matrices are used to determine the locations of the corresponding input/output degrees of freedom within the system’s total degrees of freedom. The installation matrix is used to convert disturbance forces/control forces from local coordinates to global coordinates. Since the ANC method described in Equation (17) requires the secondary path for compensation during the iteration of the weight coefficients, Figure 5b illustrates the system for identifying a single control channel through ANC itself. The output WCof satisfies the following equation:

(20) $$\begin{array}{c} WCof=\left[\begin{array}{cc} C_{sin} & C_{cos} \end{array}\right]\\ \rho \sin\left(2\pi f_{ctr}n+\varphi \right)=C_{sin}\sin\left(2\pi f_{ctr}n\right)+C_{cos}\cos\left(2\pi f_{ctr}n\right) \end{array}$$

#### 2.2.2. Optimization-Based Determination of Control Parameters

**Figure 6:**
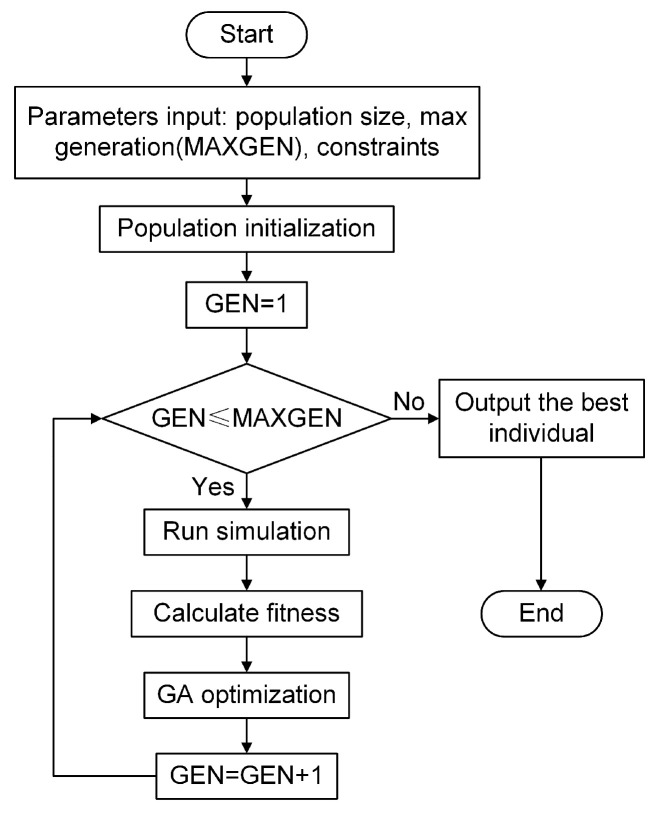
Flow chart of Simulink–GA co-optimization.

The simulation involves a total of 24 independent control parameters. The two control parameters of each channel not only determine the control performance of that channel but may also theoretically affect the control performance of the other channels. To conveniently determine all the control parameters and achieve better control performance, this study combines optimization algorithms with the simulation system for joint parameter optimization. A genetic algorithm (GA) is used as an example, and its basic process is shown in Figure 6.

In the above process, the individuals of the algorithm are 1 × 24 vectors, corresponding to the 24 control parameters that need to be determined. The purpose of running the simulation is to obtain the control process data corresponding to a specific individual. For optimization, an objective function needs to be designed based on this data. Since structural vibrations produce both positive and negative peaks, the vibration magnitude is mainly evaluated using the root mean square (RMS) value of the vibration signal over 10 cycles (approximately 0.24 s) in the simulation. Specifically, for the vibration signal $y_{j}(n)$ of the j-th channel, its RMS value is defined as:

(21) $$y_{jrms}=\sqrt{\sum_{n}^{n+239}y_{j}^{2}\left(n\right)/240}$$

Based on the definition of flatness, the RMS values of the vibration at the center points of the 12 sub-mirrors are further processed to calculate the RMS, which is used as the standard for evaluating the overall flatness of the primary mirror. On this basis, two types of values are proposed as indices for evaluating the overall vibration level of the 12 channels: (I) the RMS value of the RMS values of each of the 12 channels during the control process, expressed as

(22) $$y_{rms}=\sqrt{\frac{\sum y_{jrms}^{2}}{12}}$$

(II) the maximum RMS value among the RMS values of the channels during the control process, expressed as

(23) $$y_{max}=max\left(y_{jrms}\right)$$

**Figure 7:**
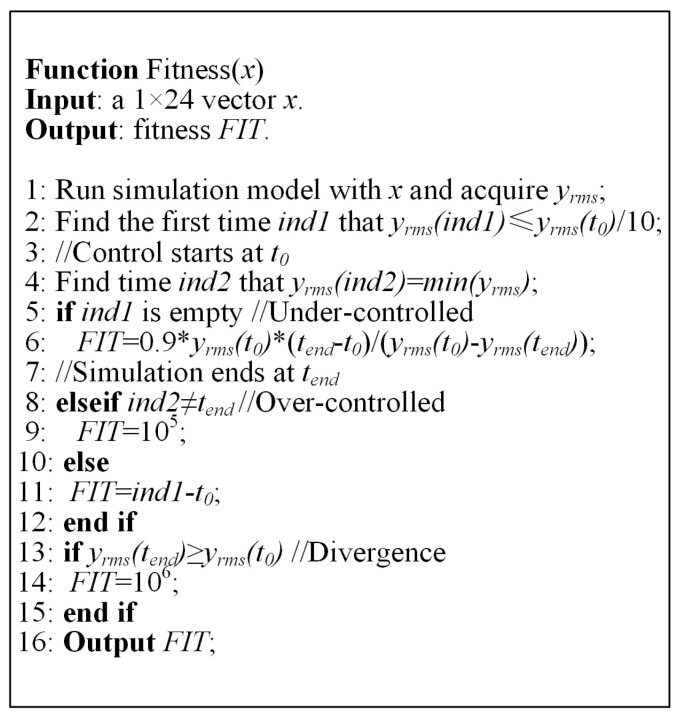
Pseudocode of fitness function.

The latter aims to ensure that the control process balances all channels and facilitates uniform and rapid convergence of each channel. In this simulation, the fitness is defined as the time it takes for the index to converge to less than 10% of its value at the start of control. A smaller fitness value indicates better control performance. To improve optimization efficiency, the Simulink simulation time is set to 8 s, with control starting at 3 s. According to the algorithm characteristics, three situations may occur in the simulation: control divergence, over-controlled (control converges and then diverges), and under-controlled (control performance is insufficient at the end of the simulation). For control divergence, the fitness is set to a large value; for over-controlled, it is set to another relatively smaller large value; for under-controlled, the fitness is based on the predicted convergence time through the average convergence rate. The pseudocode for the fitness function based on the RMS index is shown in Figure 7.

### 2.3. Experimental Setup

To verify the feasibility and effectiveness of the aforementioned attachable absorber and distributed vibration control method, prototypes of the attachable absorber and a structural model were designed, and an experimental system was built to conduct distributed vibration control tests.

**Figure 8:**
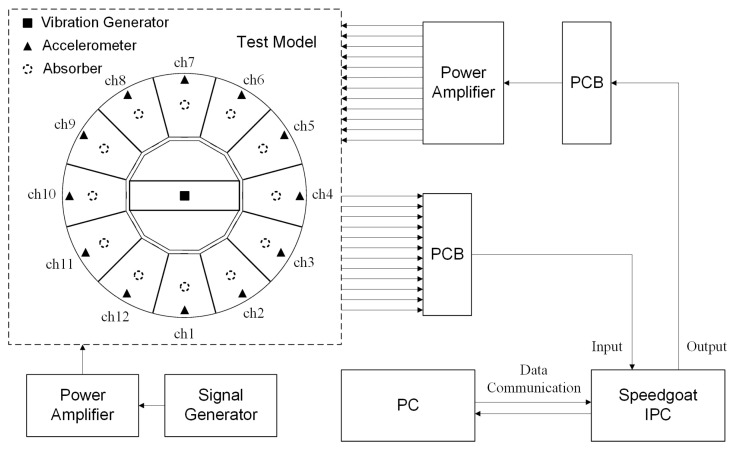
Diagram of the experimental setup.

**Figure 9:**
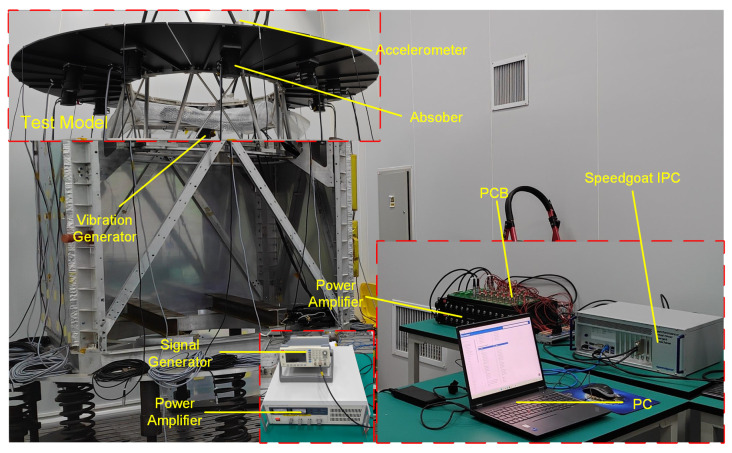
Complete experimental setup.

The experimental system mainly consists of the test structure model, data acquisition system, control system, and excitation system. The test structure model is mounted on a satellite payload bay model, with the payload bay fixed to the foundation. The structure model is made of aluminum and includes a main bracket, primary mirror, supporting rods, and secondary mirror. The primary mirror’s individual sub-mirrors are bolted onto the main bracket, with no direct contact between them, forming the subject of the test. The data acquisition system includes accelerometers, data acquisition equipment, and a test computer (PC). All 12 accelerometers are low-frequency piezoelectric accelerometers (Model 1A206E) from DONGHUA, with a sensitivity of 9800 mV/g. They are installed at the surface edges of each secondary mirror. The data acquisition equipment is an industrial PC (IPC) from Speedgoat, which relies on a high-performance FPGA module. This module supports multi-channel analog signal acquisition, processing, and output. After connecting to the test computer, it works with the real-time control module in the Simulink library to directly compile models in Simulink into code, enabling the IPC to collect accelerometer voltage signals, perform data interaction, and generate and output control voltage signals. The control system includes 12 attachable absorbers based on the design in Figure 3, a corresponding power amplifier, as well as the IPC and test computer. The 12 absorbers are installed at the central position of the bottom surface of each sub-mirror. The IPC interfaces with the sensors and amplifiers through a circuit board (PCB). The excitation system mainly consists of one absorber, a signal generator, and a driving power amplifier. The absorber, acting as the exciter, is installed at the center of the main bracket of the test model. It is actively driven by the signal generator in combination with the power amplifier to simulate a constant-frequency excitation within the payload. The system layout schematic is shown in Figure 8 and Figure 9 presents the actual on-site configuration.

The sampling rate of the control system is 1000 Hz. Before the test, each channel is identified using the method described by Equation (20), and the results are written into the control program. The test duration for each round is 60 s, and the basic process is as follows:

1. Collect noise for about 10 s;
2. Around the 10 s mark, activate the excitation system at a frequency of 42 Hz;
3. At 20 s, activate the control system to perform vibration control;
4. At 50 s, deactivate the vibration control system;
5. At 60 s, deactivate the excitation, marking the end of the round.

The testing procedure described above was adopted to more intuitively demonstrate the effects of environmental noise, disturbance excitation on/off, and vibration control on/off on the signals of each channel, thereby better reflecting the control performance. A total of 13 rounds of tests were conducted. In the first 12 rounds, only one channel was controlled sequentially (for example, in the first round, control ch1). In the 13th round, all channels were controlled simultaneously. During the 13 rounds of testing, all parameters remained unchanged except for the controlled channel.

## 3. Results

### 3.1. Frequency Response Analysis of the Absorber Model

**Figure 10:**
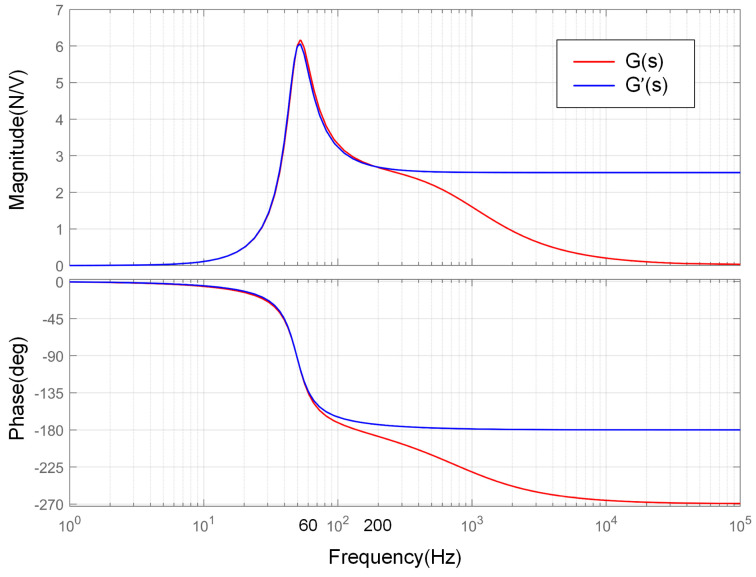
Bode diagram of the two absorber models.

The relevant parameters used in the analysis of the absorber model are shown in Table 1. By substituting the parameters from the table into the transfer function models described by Equations (8) and (9), a frequency response analysis is performed, resulting in the magnitude gain and phase delay for both models, as shown in Figure 10.

From the amplitude–frequency characteristics, it can be observed that both models exhibit curves with distinct resonance peaks, and the resonance frequency (approximately 52.2 Hz in this case) and magnitude are nearly identical. Before the resonance frequency, the amplitude–frequency and phase–frequency curves of both models overlap. As the frequency decreases, the gain rapidly diminishes to 0. The difference is that as the frequency increases, the gain of the simplified model stabilizes at a non-zero constant value and remains in anti-phase with the input, while the gain of the original model gradually approaches 0 and the phase difference stabilizes at 90°. Even so, before 200 Hz, both models still exhibit similar amplitude–frequency characteristics, with phase–frequency differences not exceeding 6°. This indicates that at relatively low frequencies, the simplified second-order system can be used as a model for the absorber.

**Figure 11:**
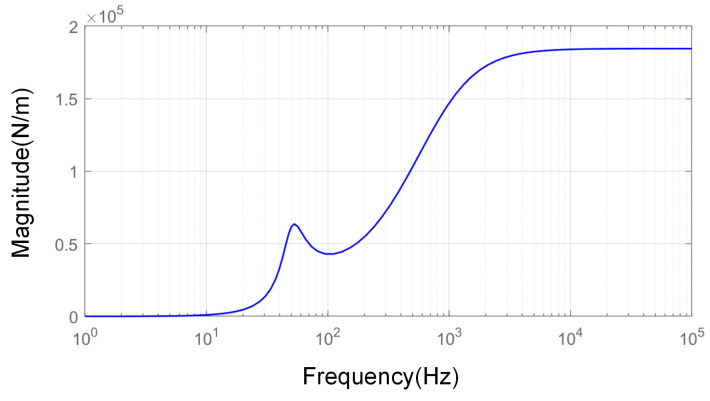
Amplitude–frequency characteristics of $F_{c}\left(s\right)$-$X_{b}\left(s\right)$.

Additionally, a frequency response analysis was also conducted for the relationship between the displacement of the objective and the output force of the absorber based on Equation (7). The amplitude–frequency curve is shown in Figure 11. Essentially, this relationship describes the constraint force exerted by the absorber on the objective when there is no control voltage input to the absorber and the displacement of the objective structure is fixed, while ignoring the various practical constraints.

In the on-orbit environment, the displacement of micro-vibrations is typically on the order of 10^−6^ m, with acceleration levels usually around 10^−3^ g. The displacement decreases as the frequency increases. Based on the above curves, the maximum force exerted is no greater than 0.1 N, and this force will further decrease with the implementation of vibration control. Therefore, it is reasonable to neglect this effect.

### 3.2. Optimization and Simulation Results and Analysis

Based on the optimization method described in the previous section, fitness functions were designed using two types of index values, and GA optimization was performed with population sizes of 200 and 500, with other parameters remaining consistent. A portion of the optimization results is shown in Table 2. Here, $t_{dm}$ represents the time required for the maximum response channel’s signal to decay by 90% after the control starts, $t_{dn}$ represents the time for the minimum response channel’s signal to decay by 90%, and $t_{d}$ represents the time for the overall flatness error $y_{rms}$ to decay by 90%. $y_{me}$ represents the maximum difference in RMS values between channels 1 s after control starts, and $y_{re}$ represents the value of $y_{rms}$ at that time. Additionally, “Generation” refers to the number of iterations where the fitness change is less than 0.001.

**Figure 12:**
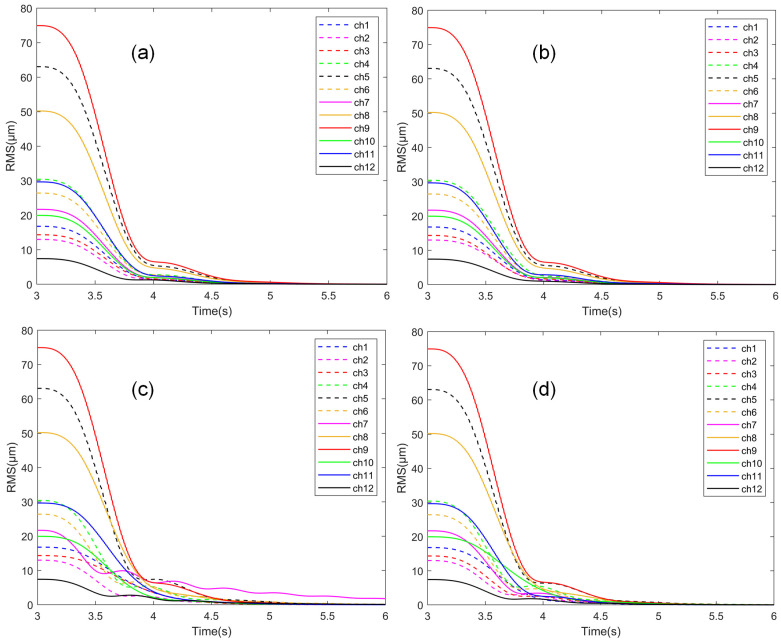
RMS convergence curves of channels under different optimization parameters: (**a**) fitness type I, 200 population; (**b**) fitness type I, 500 population; (**c**) fitness type II, 200 population; (**d**) fitness type II, 500 population.

Using the parameters obtained from the four optimization results, closed-loop control simulations were conducted. The RMS value convergence of the 12 channels (ch1~ch12) is shown in Figure 12.

The statistical data in Table 2 and the convergence curves in Figure 12 indicate that the control parameters obtained under the four optimization conditions can achieve simultaneous vibration suppression for all channels. However, the convergence processes for the parameters obtained under different conditions vary.

When using the first type of fitness function, the convergence trends of all channels remain nearly the same for control parameters obtained with different population sizes. However, there are slight differences in the control effectiveness for certain channels. For instance, in condition (a), the control effectiveness for ch2 and ch11 is better, while in condition (b), the control effectiveness for ch12 is better. From the perspective of convergence time, the control effects of parameters obtained from both conditions are almost identical, with condition (b) being slightly better. However, in terms of convergence effectiveness at 4 s, condition (a) performs slightly better. The relative error between the two is small, with a relative error of about 5.4% for $y_{me}$ and about 1.5% for $y_{re}$. Given that the LMS algorithm is inherently robust to convergence coefficients and that GA algorithm-generated individuals have a degree of randomness, such errors are inevitable. Therefore, it can be concluded that the parameters optimized with both population sizes yield nearly identical control performance.

When using the second type of fitness function, there is a noticeable difference in the control effectiveness of the control parameters obtained with different population sizes. In condition (c), the control performance for ch7 is significantly worse, and after 4 s, it even becomes the new channel with the maximum output. Since the final fitness is determined as 0.92 s, which corresponds to the maximum value before 4 s, the convergence disadvantage of ch7 during this period is not detected, suggesting that the algorithm might have become trapped in a local optimum. This issue was mitigated by increasing the population size, as seen in condition (d), where the convergence uniformity across channels improved significantly. Various indicators reflecting the convergence effectiveness at 4 s were noticeably better in condition (d) compared to condition (c).

A comparison of the optimization results for the two different types of fitness functions reveals that although the second fitness function can indeed reduce the differences between channels during the vibration control process and improve uniformity, its final control effectiveness is not as good as that of the first function. Furthermore, when the population size is insufficient, it is more prone to local optima. The above results also indicate that, with an appropriate fitness function, the optimization of distributed ANC controller parameters does not overly rely on the population size, even when the number of control parameters is large.

### 3.3. Influence of the Coupling of the Controller

**Figure 13:**
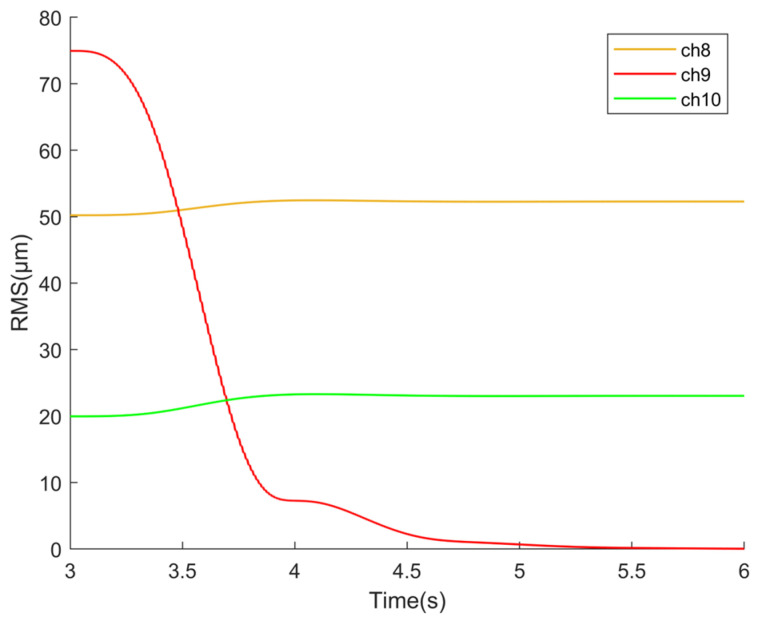
Influence of ch9’s control on ch8 and ch10.

As mentioned before, since the objective is essentially a MIMO system, there is inherent coupling between the control channels, and the control signal of a single channel can introduce additional input to other channels. Additionally, the convergence process of the LMS algorithm is related to the input signal, so the control parameters of different channels in the distributed ANC controller may also exhibit coupling. In condition (a), parameters are applied to control only channel ch9, and the output changes in this channel as well as its neighboring channels, ch8 and ch10, are observed. As shown in Figure 13, when controlling ch9, the steady-state signals of ch8 and ch10 increase by approximately 4.1% and 15.4%, respectively.

To investigate the impact of this coupling and its implications for the optimization strategy, a comparative simulation was conducted by adding single-channel control parameter optimization. The comparison strategy was as follows: based on the optimization strategy from the previous section, two control parameters for each channel were optimized separately using the type I fitness function, focusing only on the output of the current channel. A total of 12 sets of 24 control parameters were obtained, which were then used for single-channel and full-channel control simulations. The convergence behavior and overall indices for each channel under three control conditions were compared and analyzed:

- Case 1: using the parameters from condition (a) in the previous section for full-channel control;
- Case 2: using the parameters from this section for full-channel control;
- Case 3: using the parameters from this section for individual control of each channel, with other channels not controlled, but summarizing the overall indices based on the control process of each channel.

**Figure 14:**
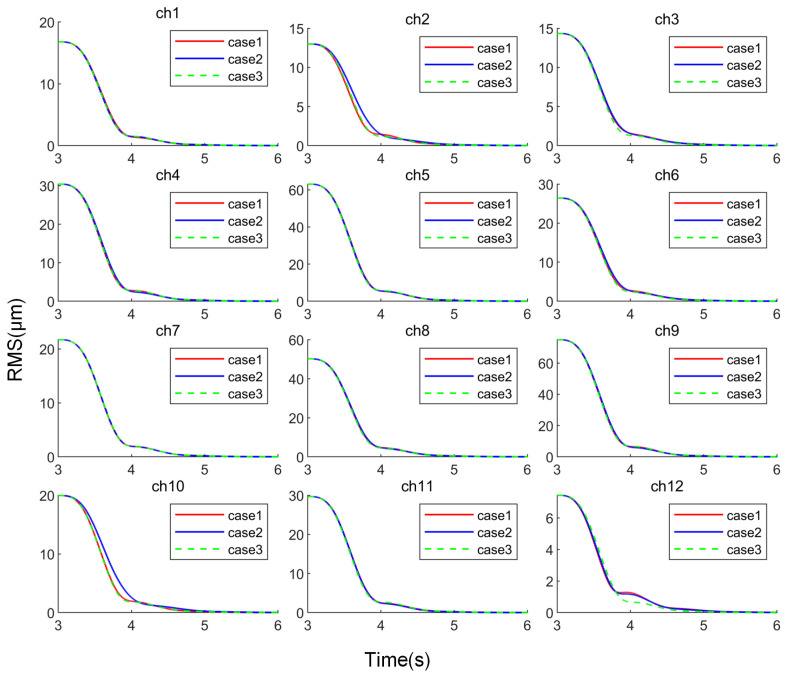
RMS convergence curves of channels under different cases.

The partial data statistics of the simulation results for the three cases are shown in Table 3, and the convergence of each channel is shown in Figure 14. It should be noted that since simultaneous control of all channels is required in practice, the relevant data for Case 3 were only used for analysis and comparison, and could not be implemented.

By observing the convergence curves of each channel in Figure 14, it can be seen that the convergence processes of most channels are almost identical under the three control conditions, with only ch2, ch10, and ch12 showing noticeable differences. For ch2, the control effects after 4 s under the three cases are almost the same, but Case 2 exhibits a more uniform convergence speed before 4 s, although the convergence effect is slightly worse than the other two cases. Ch10 shows the same pattern, with the convergence process becoming consistent around 4.1 s for all three cases. For ch12, Case 2 and Case 1 display consistent convergence characteristics, and Case 3 shows better convergence performance before the signal completely converges. From Table 3, it can be seen that Case 3 achieves better convergence speed, especially for channels with smaller output signals. However, in terms of final control effects, there is no significant difference between the three cases, except that Case 3, with faster convergence of small output signals, results in a larger maximum difference in output across channels.

The above results show that for the distributed ANC controller in this study, even when the parameters for each channel are optimized independently, the obtained parameters can still be used for full-channel control and maintain convergence. A comparison between Case 2 and Case 3 reveals that the impact of system coupling does indeed exist. Under the same parameters, the coupling causes changes in the convergence process for some channels compared to independent control, with this effect being more noticeable in channels with smaller output signals. Although the coupling slightly reduces the overall convergence speed, it has almost no effect on the final control performance. A comparison between Case 1 and Case 2 shows that there is no significant difference in the convergence process or final control performance between the two. Considering parameter optimization errors, the results can also be interpreted as showing equivalent control performance. This indicates that the system’s coupling does not significantly affect the parameter optimization method described earlier. The parameter optimization itself can also be decoupled, meaning that each channel can be optimized independently based on its individual control performance, and the final result will be the same as that achieved through global parameter optimization.

The actual large-scale space payload structures are complex and may involve a large number of vibration control points. Adjusting the parameters for all these control channels simultaneously is quite challenging. Based on the above discussion, it can be concluded that when using the aforementioned ANC controller, independently tuning the parameters for each control channel can still achieve the desired final control objectives.

### 3.4. Experimental Results and Analysis

**Figure 15:**
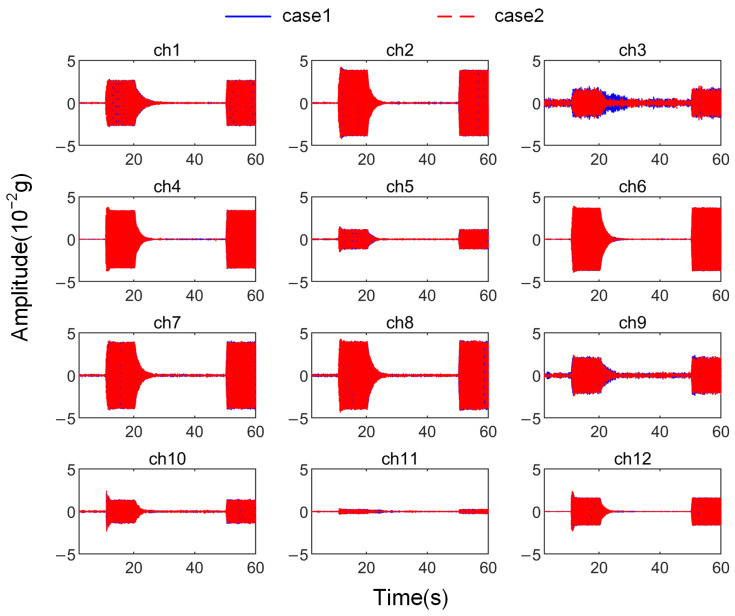
Signals of all channels in different cases during the test.

In the experiment, the situation where all channels are controlled is was referred to as Case 1, and the situation where only one channel is controlled is was referred to as Case 2. The time-domain signal changes corresponding to all channels in both cases are shown in Figure 15.

**Figure 16:**
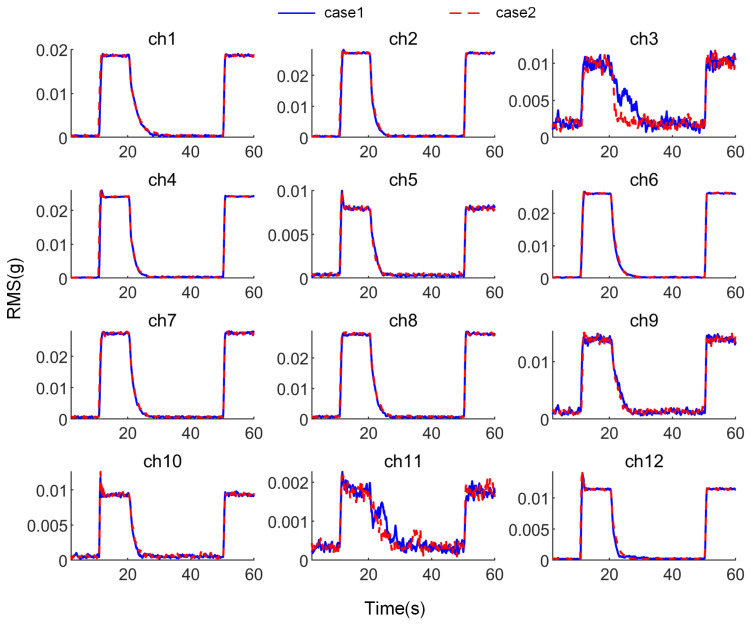
RMS of signals of all channels during the test.

Correspondingly, the RMS value changes for each channel are shown in Figure 16.

Using the RMS value as the standard, the above results are statistically analyzed. Since noise is present in the actual test, the following definitions are made: for all channels, the RMS values during noise, disturbance, and steady convergence are denoted as $R_{n}$, $R_{d}$, and $R_{c}$, respectively, with all values representing the average levels during steady states. The convergence rate $P_{c}$ is defined as

(24) $$P_{c}=\frac{R_{d}-R_{t}}{R_{d}-R_{n}}$$

The real-time RMS value is denoted as $R_{t}$. The final convergence rate, $P_{ce}$, is defined as the convergence rate when $R_{t}=R_{c}$. $t_{c0.95}$ is the time required for the convergence rate to stabilize above 0.95, and $t_{c0.9}$ is the time required for the convergence rate to stabilize above 0.9. All these times are referenced from the start of control (i.e., from 20 s). Based on the above definitions, the statistical results of the test for the 12 channels under both conditions are shown in Table 4.

As shown in Figure 15 and Figure 16, when distributed vibration control is applied using the attachable absorber with the distributed ANC controller described earlier, simultaneous control of vibration signals from all 12 channels is achieved. The data in Table 4 indicate that the signal convergence rate of all channels exceeds 0.95, bringing the vibration level down to the ambient noise level. It is important to note that the final convergence rate of some channels exceeds 1, mainly due to noise interference. On the one hand, the 42 Hz component mixed in the noise is also suppressed; on the other hand, during the convergence process, noise fluctuations can cause the noise level to drop below the level at the start of testing.

Compared to the case of controlling a single channel, the convergence process for most channels in the distributed control scenario is almost identical to the process when they are controlled individually, with the variation in the convergence indices being within the error range. However, ch3 and ch11 show significant differences, which require further discussion.

## 4. Discussion

The issue with channel ch11 is relatively clear. As observed from the previous results, the response of ch11 after the disturbance is activated is the smallest among all channels, resulting in a lower signal-to-noise ratio for this channel. The RMS noise level is approximately 18.9% of the disturbance level. The presence of noise affects both the convergence speed of the LMS algorithm and the convergence rate of ch11, which is largely determined by the noise. Although the RMS level of the noise is defined by averaging, it remains a random signal, which introduces randomness into the final convergence state of ch11 as defined by the ratio. This is reflected in Table 4, where the convergence time is longer, and the relationship between $t_{c0.9}$ and $t_{c0.95}$ is inconsistent and exhibits significant differences.

**Figure 17:**
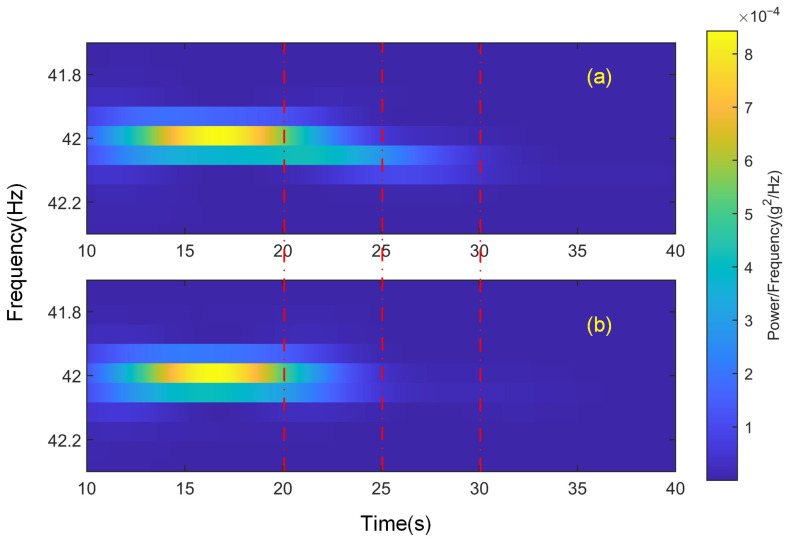
Power spectrum with time of ch3 signal in different cases: (**a**) Case 1; (**b**) Case 2.

The situation with channel ch3 is relatively more complex. First, it can be noted that the noise level of ch3 is the highest among all channels, almost an order of magnitude higher than the others. However, its response after the disturbance is activated is moderate, resulting in the lowest signal-to-noise ratio, with the RMS noise level being approximately 20% of the disturbance level. This causes ch3 to encounter similar issues to those observed in ch11 during the test. More importantly, the relatively high noise level also indicates that this part of the structure is more susceptible to various environmental disturbances, making it more likely to respond to control forces applied by other channels. To better understand the signal variation in this channel during the test, the raw time-domain signals of the channel under both cases are subjected to short-time Fourier transform (STFT), providing the time-varying power spectrum of the signal, as shown in Figure 17.

Figure 17 illustrates the time-varying energy distribution of different frequency components in the ch3 signal. From 10 s to 20 s, Figure 17a,b show identical energy distributions, corresponding to the activation of the disturbance during the test. After 20 s, as the control begins, the energy at 42 Hz starts to attenuate. It can be seen that from 20 s to 22 s, the energy at 42 Hz in Figure 17a is slightly higher than that in Figure 17b, because, at this point, the 42 Hz control signal from other channels partially affects ch3. However, by 25 s, the energy at 42 Hz in both cases has effectively attenuated, and the energy in Figure 17a is even slightly lower. At this point, ch3 in Case 2 has almost reached steady convergence. It is important to note that just above 42 Hz, there is another frequency component, $f_{\delta }$, whose energy is significantly higher than the other components. In Figure 17b, the energy of $f_{\delta }$ begins to decay gradually before the control starts (around 20 s), with a slow decay rate. After 25 s, it becomes the dominant component. In contrast, in Figure 17a, the energy of $f_{\delta }$ increases after 20 s, peaking around 23 s, and then starts to decay. This is the primary reason for the slower convergence of ch3 in Case 1. Only after 30 s, when the energy of $f_{\delta }$ decreases to a low level, does ch3 reach steady convergence, which is consistent with the trends observed in Figure 15 and Figure 16, and Table 4.

**Figure 18:**
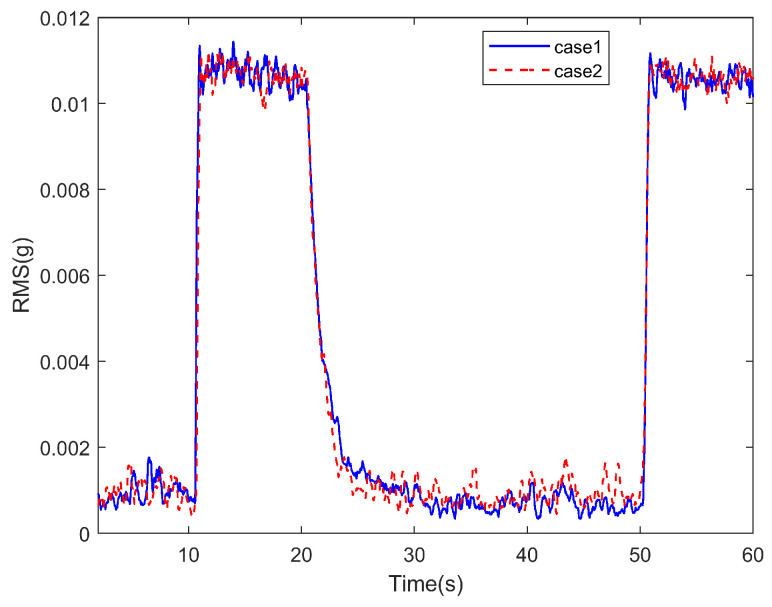
Signal of ch3 in different cases with 84 Hz disturbance.

The most likely cause of this phenomenon is that there exists a local mode in the submirror structure around 42 Hz, where the frequency components are more densely concentrated. The control forces from other sub-mirrors caused an instantaneous response in this mode, further increasing the energy at this frequency, which required more time to attenuate. This phenomenon only appears at specific frequencies. As shown in Figure 18, a similar test was conducted under 84 Hz excitation, with bandpass filtering applied to the input signal in the program to reduce noise. The results show no significant difference in the ch3 signal between the two cases.

The structural model used in this test is primarily for conceptual verification, and its design, manufacturing, and installation processes are relatively crude, which may have contributed to the observed phenomenon. In actual space payloads, the design must ensure that the structure’s fundamental frequency avoids typical disturbance frequencies to prevent such phenomena from occurring.

**Figure 19:**
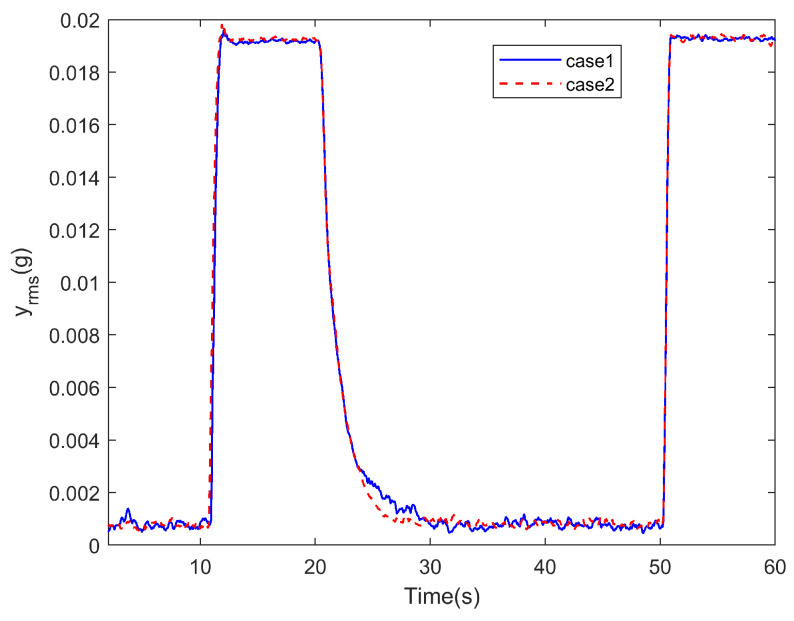
$y_{rms}$ of all channels’ signals in different cases.

Figure 19 shows the variation of the total RMS value $y_{rms}$, as defined in the previous section, with related statistical data provided in Table 5. The figure demonstrates that the distributed vibration control method used in the test can simultaneously control the vibration of submirrors, reducing the normal position deviation of each point on the primary mirror. This ensures that the primary mirror maintains its flatness even under disturbances. A comparison of the convergence processes under the two cases shows that, although differences in the convergence of some channels were observed earlier, their impact on the overall flatness variation is minimal. The coupling effect during multi-channel simultaneous control only affects the convergence speed near the steady-state phase, without impacting the final convergence outcome. In this test, compared to the case where the submirrors are entirely independent, the coupling effect reduces the final-stage convergence speed by approximately 10%, with the actual time difference being within 1 s.

## 5. Conclusions

This paper proposes a method for distributed vibration control of large space payload structures using attachable absorbers. It involves related modeling, simulation, and experiments, mainly including the following: (1) the absorber was modeled and analyzed, indicating that its voltage–force output in the low-frequency range can be simplified as a second-order system; (2) a combined Simulink–GA optimization approach was used to handle the large number of convergence parameters during simulation; (3) further optimization was conducted, indicating that the coupling between control channels slightly reduced the convergence speed but did not impact the final control effect; thus, this controller can achieve the same effect by independently adjusting the parameters of each channel as by adjusting all parameters simultaneously; and (4) a distributed vibration control experiment was conducted based on a physical testing system. The test results show that the proposed method can simultaneously control the vibration of the sub-mirrors.

The contributions of this paper are as follows: (1) a distributed micro-vibration control method based on distributed active vibration absorbers and a combined ANC controller is proposed, and its practical effectiveness is validated through the experiment; (2) the complete modeling process of the system, including the actuators, is presented, and a parameter optimization approach is proposed to address the issue of selecting multiple control parameters; and (3) the comparative analysis of different optimization strategies is conducted to evaluate the impact of system coupling on the control effects of the controller’s predetermined parameters, providing theoretical support and guidance for engineering practice.

## Figures and Tables

**Table 1:** Analytical parameters of the absorber.

Item	Unit	Value
$m_{a}$	g	243
$k_{a}$	N/m	23,256
$k_{e}$	N/A	12.7
R	Ω	5
L	mH	1

**Table 2:** Partial statistics of optimization and simulation results.

Fitness Type	Population	Generation	Final Fitness	$t_{dm}$ (s)	$t_{dn}$ (s)	$t_{d}$ (s)	$y_{me}$ (μm)	$y_{re}$ (μm)
I	200	33	0.935	0.922	1.260	0.942	5.33	3.35
	500	26	0.935	0.922	1.200	0.942	5.62	3.40
II	200	13	0.920	0.920	1.542	1.181	5.52	4.76
	500	13	0.919	0.919	1.244	1.093	5.08	4.18

**Table 3:** Partial simulation results of the cases.

Case	$t_{dm}$ (s)	$t_{dn}$ (s)	$t_{d}$ (s)	$y_{me}$ (μm)	$y_{re}$ (μm)
1	0.922	1.260	0.942	5.33	3.35
2	0.930	1.254	0.946	5.11	3.29
3	0.923	0.929	0.924	6.12	3.34

**Table 4:** Statistics of the test.

ch	Case	$R_{n}$ (g)	$R_{d}$ (g)	$R_{c}$ (g)	$P_{ce}$	$t_{c0.9}(s)$	$t_{c0.95}$ (s)
1	1	0.00040	0.0186	0.00041	0.999	5.5	6.7
	2	0.00040	0.0186	0.00044	0.998	5.5	7.4
2	1	0.00042	0.0272	0.00050	0.997	3.3	4.4
	2	0.00043	0.0272	0.00044	1.000	3.6	4.8
3	1	0.0020	0.0100	0.0019	1.005	9.4	9.5
	2	0.0019	0.0099	0.0019	1.004	4.3	4.9
4	1	0.00021	0.0240	0.00038	0.993	3.3	4.3
	2	0.00019	0.0240	0.00025	0.998	3.4	4.7
5	1	0.00039	0.0080	0.00032	1.010	3.6	4.0
	2	0.00039	0.0079	0.00041	0.997	3.4	4.4
6	1	0.00027	0.0260	0.00030	0.999	4.0	5.2
	2	0.00027	0.0261	0.00035	0.997	4.2	5.0
7	1	0.00062	0.0272	0.00065	0.999	3.8	4.5
	2	0.00059	0.0273	0.00067	0.997	4.0	5.2
8	1	0.00074	0.0276	0.00065	1.003	3.7	4.6
	2	0.00068	0.0280	0.00077	0.997	4.2	4.9
9	1	0.00120	0.0138	0.00130	0.997	4.9	5.7
	2	0.00120	0.0139	0.00140	0.986	4.2	5.6
10	1	0.00052	0.0093	0.00055	0.997	3.2	3.3
	2	0.00053	0.0093	0.00064	0.988	3.5	4.3
11	1	0.00034	0.0018	0.00032	1.012	9.1	11.1
	2	0.00033	0.0018	0.00037	0.972	7.0	16.6
12	1	0.00021	0.0114	0.00023	0.998	2.6	3.0
	2	0.00021	0.0114	0.00025	0.996	3.5	4.5

**Table 5:** Statistics of $y_{rms}$.

Case	$R_{n}$ (g)	$R_{d}$ (g)	$R_{c}$ (g)	$P_{ce}$	$t_{c0.9}(s)$	$t_{c0.95}$ (s)
1	0.00079	0.0192	0.00079	1.000	4.4	9.1
2	0.00077	0.0192	0.00081	0.997	4.0	8.1

## Data Availability

The raw data supporting the conclusions of this article will be made available by the authors on request.

## References

[1] McElwain M.W., Feinberg L.D., Perrin M.D., Clampin M., Mountain C.M., Lallo M.D., Lajoie C.-P., Kimble R.A., Bowers C.W., Stark C.C. (2023). The James Webb Space Telescope Mission: Optical Telescope Element Design, Development, and Performance. Publ. Astron. Soc. Pac..

[2] Barto A.A., Atkinson C., Contreras J., Lightsey P.A., Noecker C., Waldman M., Whitman T., Oschmann J.M., De Graauw M.W.M., MacEwen H.A. (2008). Optical Performance Verification of the James Webb Space Telescope.

[3] Chen Z., Wang G., Li J. (2024). Acceleration Function Evaluation and Selection for On-Orbit Moving Mechanism Based on Spectral Analysis. J. Vib. Eng. Technol..

[4] Haghshenas J. (2017). Vibration Effects on Remote Sensing Satellite Images. Adv. Aircr. Spacecr. Sci..

[5] Zhou X., Liu H., Li Y., Ma M., Liu Q., Lin J. (2021). Analysis of the Influence of Vibrations on the Imaging Quality of an Integrated TDICCD Aerial Camera. Opt. Express.

[6] Chen Z., Wang G., Li J., Liu F., Wu Y., Wang D. (2024). Modeling and Experimental Investigation of an Active Angular Vibration Absorber. Int. J. Str. Stab. Dyn..

[7] Ni T.Z., Yang B.Y. (2017). Adaptive Active Vibration Control Method for Cryocooler Compressor in Space. Chin. Space Sci. Technol..

[8] Jiao X., Zhang J., Li W., Wang Y., Ma W., Zhao Y. (2023). Advances in Spacecraft Micro-Vibration Suppression Methods. Prog. Aerosp. Sci..

[9] Xing W., Tuo W., Li X., Wang T., Yang C. (2024). Micro-Vibration Suppression and Compensation Techniques for in-Orbit Satellite: A Review. Chin. J. Aeronaut..

[10] Hanagan L.M., Kulasekere E.C., Walgama K.S., Premaratne K. (2000). Optimal Placement of Actuators and Sensors for Floor Vibration Control. J. Struct. Eng..

[11] Pereira E., Díaz I.M., Hudson E.J., Reynolds P. (2014). Optimal Control-Based Methodology for Active Vibration Control of Pedestrian Structures. Eng. Struct..

[12] Camacho-Gómez C., Wang X., Pereira E., Díaz I.M., Salcedo-Sanz S. (2018). Active Vibration Control Design Using the Coral Reefs Optimization with Substrate Layer Algorithm. Eng. Struct..

[13] Cao Y., Cao D., Huang W. (2019). Dynamic Modeling and Vibration Control for a T-Shaped Bending and Torsion Structure. Int. J. Mech. Sci..

[14] Swanson D., Black P., Girondin V., Bachmeyer P., Jolly M. (2015). Active Vibration Control Using Circular Force Generators. https://data.epo.org.

[15] Tang Y.Z., Lu G.Y., Cai G.P. (2022). Active Vibration Control of Truss Structures for Large Space Telescopes Based on Cable Actuators. Appl. Math. Mech..

[16] Tzou H.S., Tseng C.I. (1991). Distributed Vibration Control and Identification of Coupled Elastic/Piezoelectfuc Systems: Finite Element Formulation and Applications. Mech. Syst. Signal Process..

[17] Burke S., Hubbard J. (1987). Active Vibration Control of a Simply Supported Beam Using a Spatially Distributed Actuator. IEEE Control Syst. Mag..

[18] Berkhoff A.P., Nijsse G. (2007). A Rapidly Converging Filtered-error Algorithm for Multichannel Active Noise Control. Adapt. Control Signal.

[19] Berkhoff A.P., Wesselink J.M. (2011). Combined MIMO Adaptive and Decentralized Controllers for Broadband Active Noise and Vibration Control. Mech. Syst. Signal Process..

[20] Kumar R., Singh S.P., Chandrawat H.N. (2007). MIMO Adaptive Vibration Control of Smart Structures with Quickly Varying Parameters: Neural Networks vs. Classical Control Approach. J. Sound Vib..

[21] Li T., Wang Z., Li J., Ma Y. (2013). Distributed Vibration Control of Tensegrity Structure. J. Vib. Control.

[22] Ferrari G., Amabili M. (2015). Active Vibration Control of a Sandwich Plate by Non-Collocated Positive Position Feedback. J. Sound Vib..

[23] Omidi E., Mahmoodi S.N. (2016). Vibration Suppression of Distributed Parameter Flexible Structures by Integral Consensus Control. J. Sound Vib..

[24] Wang E., Wu S., Liu Y., Wu Z., Liu X. (2019). Distributed Vibration Control of a Large Solar Power Satellite. Astrodynamics.

[25] Wang E., Wu S., Wu Z., Radice G. (2019). Distributed Adaptive Vibration Control for Solar Power Satellite during On-Orbit Assembly. Aerosp. Sci. Technol..

[26] Li C., Chen Z., Zu H., Zhao Y. (2018). An Improved Optimal Adaptive Control Method for MIMO Sine Vibration Control of a Multichannel Coupled System. Proceedings of the Volume 11: Acoustics, Vibration, and Phononics.

[27] Xie Y., Shi H., Bi F., Shi J. (2018). A MIMO Data Driven Control to Suppress Structural Vibrations. Aerosp. Sci. Technol..

[28] Ji N., Liu J. (2020). Distributed Vibration Control for Flexible Spacecraft with Distributed Disturbance and Actuator Fault. J. Sound Vib..

[29] Shan G., Li Y., Zhang L., Wang Z., Zhang Y., Qian J. (2015). Contributed Review: Application of Voice Coil Motors in High-Precision Positioning Stages with Large Travel Ranges. Rev. Sci. Instrum..

[30] Wu Y., Wang G., Yu K., Ge M., Gao X., Wang D. A Modified Fxlms Algorithm for Active Control of Parallel Multi-Frequency Narrowband Structural Vibrations. Proceedings of the 29th International Congress on Sound and Vibration.

